# Dissecting the Tectal Output Channels for Orienting and Defense Responses

**DOI:** 10.1523/ENEURO.0271-20.2020

**Published:** 2020-09-29

**Authors:** Kaoru Isa, Thongchai Sooksawate, Kenta Kobayashi, Kazuto Kobayashi, Peter Redgrave, Tadashi Isa

**Affiliations:** 1 Department of Developmental Physiology, National Institute for Physiological Sciences, Okazaki 444-8585, Japan; 2 Department of Neuroscience, Graduate School of Medicine, Kyoto University, Kyoto 606-8501, Japan; 3 Department of Pharmacology and Physiology, Faculty of Pharmaceutical Sciences, Chulalongkorn University, Bangkok 10330, Thailand; 4 Section of Viral Vector Development, National Institute for Physiological Sciences, Okazaki 444-8585, Japan; 5 Department of Life Sciences, the Graduate University for Advanced Studies (SOKENDAI), Hayama 240-0193, Japan; 6 Department of Molecular Genetics, Institute of Biomedical Sciences, Fukushima Medical University, Fukushima 960-1295, Japan; 7 Department of Psychology, The University of Sheffield, Sheffield S1 2LT, United Kingdom; 8 Institute for the Advanced Study of Human Biology (WPI-ASHBi), Kyoto University, Kyoto 606-8501, Japan; 9 Human Brain Research Center, Graduate School of Medicine, Kyoto University, Kyoto 606-8507, Japan

**Keywords:** escape, innate behavior, mouse, optogenetics, orienting, superior colliculus

## Abstract

Electrical stimulation and lesion experiments in 1980’s suggested that the crossed descending pathway from the deeper layers of superior colliculus (SCd) controls orienting responses, while the uncrossed pathway mediates defense-like behavior. To overcome the limitation of these classical studies and explicitly dissect the structure and function of these two pathways, we performed selective optogenetic activation of each pathway in male mice with channelrhodopsin 2 (ChR2) expression by Cre driver using double viral vector techniques. Brief photostimulation of the crossed pathway evoked short latency contraversive orienting-like head turns, while extended stimulation induced body turn responses. In contrast, stimulation of the uncrossed pathway induced short-latency upward head movements followed by longer-latency defense-like behaviors including retreat and flight. The novel discovery was that while the evoked orienting responses were stereotyped, the defense-like responses varied considerably depending on the environment, suggesting that uncrossed output can be influenced by top-down modification of the SC or its target areas. This further suggests that the connection of the SCd-defense system with non-motor, affective and cognitive structures. Tracing the whole axonal trajectories of these two pathways revealed existence of both ascending and descending branches targeting different areas in the thalamus, midbrain, pons, medulla, and/or spinal cord, including projections which could not be detected in the classical studies; the crossed pathway has some ipsilaterally descending collaterals and the uncrossed pathway has some contralaterally descending collaterals. Some of the connections might explain the context-dependent modulation of the defense-like responses. Thus, the classical views on the tectal output systems are updated.

## Significance Statement

The superior colliculus (SC), a conserved structure in the vertebrate brain, controls innate behaviors, including orienting and defense responses through multiple output channels. Here, we used a double viral vector technique in mice to enable selective optogenetic activation of the classical crossed and uncrossed output pathway from the deeper collicular layers. Extended optical stimulation of the crossed pathway induced contraversive head/body turns, while stimulation of the uncrossed pathway elicited defense-like behaviors, including retreat and flight. The latter responses could vary considerably depending on the environment in which the animals were tested. The two pathways possess different patterns of bilateral axonal trajectories. These results present an updated view on the structure and function of the orienting and defense pathways from the SC.

## Introduction

The midbrain superior colliculus (SC) is an evolutionary ancient brainstem center controlling the initial, rapid, sensory-driven appetitive or defensive movements that are essential for survival in the natural environment (Dean et al., 1989). Its superficial layers (SCs) receive glutamatergic visual inputs directly from the retina (Isa et al., 1998) or indirectly from the visual cortex. Efferent projections from the SCs are directed to the visual thalamus, nucleus parabigeminalis, or to deeper layers of the SC (SCd; Lane et al., 1974; Sherk, 1979; Bennett-Clarke et al., 1989; Isa et al., 1998; Lee et al., 1997; Doubell et al., 2003; Gale and Murphy, 2014). The SCd receives non-visual sensory inputs and also motor or attention-related signals from forebrain regions (May, 2006). The SCd neurons integrate information from these multiple sources and send motor commands to the brainstem and spinal cord (Sparks and Hartwich-Young, 1989), biologically salient visual cue signals to the midbrain dopamine neurons (Comoli et al., 2003; McHaffie et al., 2006; May et al., 2009), fear-related signals to the periaqueductal gray (Evans et al., 2018), and ascending motor efference copy signals to the thalamus (Sommer and Wurtz, 2006). In terms of behavioral control, previous studies in rodents suggested there are two major output channels from the SC to the brainstem (Sahibzada et al., 1986; Dean et al., 1989). One crosses the midline in the ventral tegmental decussation and projects caudally via the predorsal bundle (PDB) to the contralateral medial ponto-medullary reticular formation (PMRF) and upper cervical spinal cord. In rodents, this pathway mainly originates from the lateral part of the SC that represents the lower half of contralateral visual field, and controls orienting and approach behavior (Dean et al., 1986). In carnivores and primates, saccadic eye movements and orienting head movements are controlled by this system (Sparks and Hartwich-Young, 1989; Isa and Sasaki, 2002; May, 2006). Hereafter, we tentatively term these neurons “crossed (orienting) pathway neurons.” The ipsilateral descending projection originates mainly from neurons in the rostro-medial part of rodent SC, which represents the upper visual field. It projects caudally to the cuneiform nucleus (CnF) and ventrolateral PMRF (Redgrave et al., 1987, 1988). This pathway is believed to control defense reactions, including freezing and escape behaviors (Sahibzada et al., 1986). Hereafter, we tentatively term these neurons “uncrossed (defense) pathway neurons.” Previous studies investigating these descending SC pathways used electrical stimulation and lesion techniques combined with standard anatomical tracing and c-fos immunohistochemistry, to verify the location of activated neurons (Redgrave et al., 1987; Dean et al., 1989; King et al., 1996; Comoli et al., 2003). However, functional analyses using these traditional, but cellularly nonselective techniques, runs potential risks of activating or lesioning passing fibers from other projections, coupled with possible problems of post-lesion neuroplasticity. Furthermore, it has also been difficult to stimulate either the SCs or SCd neurons separately without affecting the other population.

The present study sought to overcome these limitations and investigate in more detail the cellular identity of orienting and defense systems in the SC and how they might be influenced by behavioral context. To this end, we tested selective activation of the individual SC output pathway neurons by a combination of two viral vectors. One is the highly efficient retrograde gene transfer vector (Kato et al., 2011) carrying Cre, while the other is the adeno-associated viral (AAV) vector carrying channelrhodopsin 2 (ChR2) downstream of a double flox sequence. This combination enabled a pathway-specific expression of ChR2. These procedures allowed us to activate each of the two SC-brainstem output channels independently. Furthermore, this double viral vector technique can reveal the morphology of the manipulated neurons, including cell bodies and axonal arborization, as shown in previous studies using similar constructs (Kinoshita et al., 2012; Sooksawate et al., 2013; Wahl et al., 2014; Ishida et al., 2016; Asboth et al., 2018). The present investigation has revealed novel aspects of the two SC output systems; presence or absence of context dependency of the induced behavior, and clear pictures of their whole axonal trajectory which indicated that some collateral branches were missed in the previous studies.

## Materials and Methods

### Animals

Forty-eight 6- to 10-week-old male C57BL/6 mice were used in this study. Condition of animal housing was the group housing until the optic fiber implantation. After the fiber implantation we chose individual housing in the transparent cage with enrichment and monitored the body weight. The Zeitgeber time is 12/12 h light/dark cycle. The experimental protocol for the use of animals was conducted following the Guidelines of the National Institutes of Health and the Ministry of Education, Culture, Sports, Science and Technology (MEXT) of Japan and was approved by the Institutional Animal Care and Use Committee of the National Institutes of Natural Sciences and Kyoto University. We made all attempts to minimize the stress, distress and number of animals used.

### Plasmid construction

**Figure 1.**
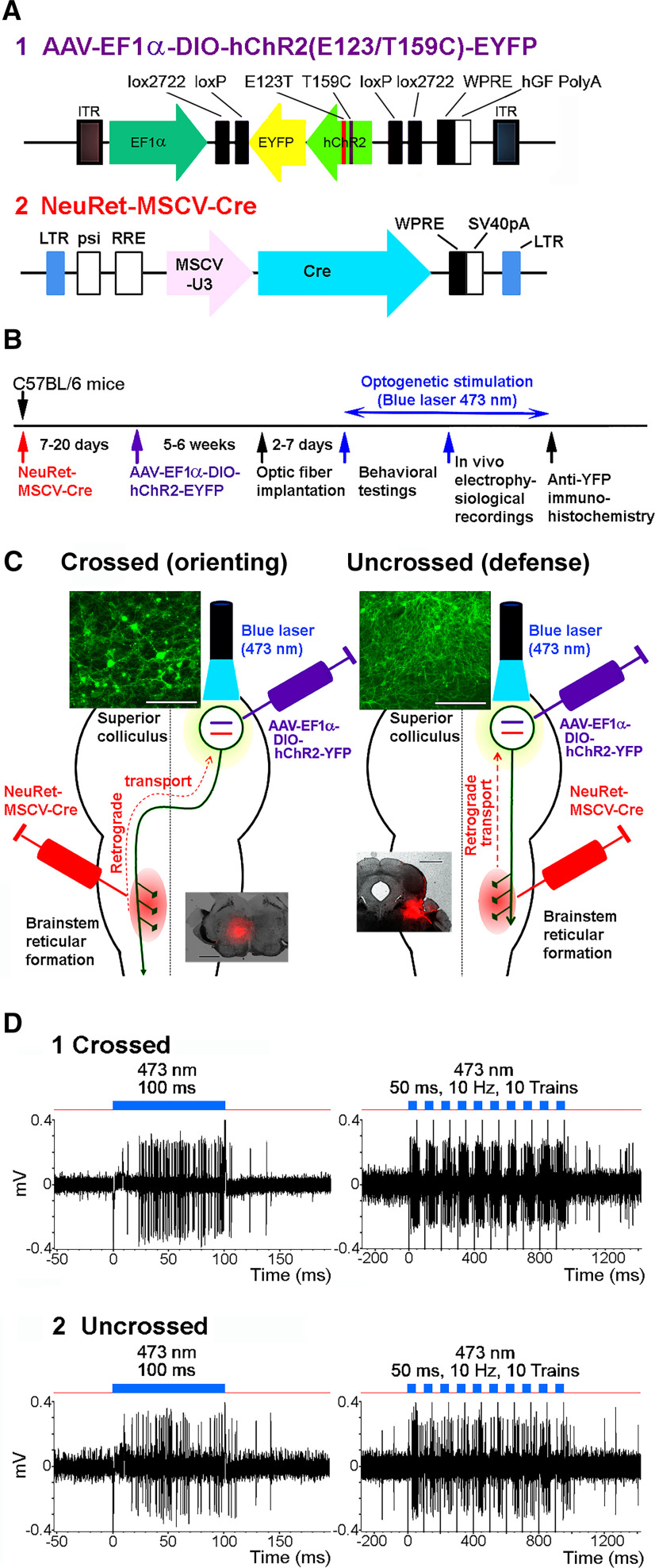
Pathway-selective optogenetic activation of SC output neurons. ***A***, Viral vector constructions. ***B***, Experimental protocols. ***C***, Schematic diagram for the double injection of the viral vectors into the brainstem and SC, and the interaction of NeuRet-MSCV-Cre and AAV-EF1α-DIO-hChR2(E123T/T159C)-EYFP in the double infected SC neurons. Upper insets in left and right panels, Photomicrographs of the somata of the crossed (orienting) and uncrossed (defense) SC-brainstem pathway neurons, respectively. Lower insets in left and right panels, Injection sites of the NeuRet-MSCV-Cre, indicated by a mock injection of Fluoro-Ruby into the brainstem reticular formation at medial PMRF and CnF, respectively. Scale bar in the upper insets = 100 μm. Scale bar in the lower insets = 1 mm. ***D***, Responses of a mouse SC output neuron to blue laser stimulation (blue line) with a 500-μm diameter fiber. (1) Responses of the crossed (orienting) pathway neurons. (2) Responses of uncrossed (defense) pathway neurons. *Figure Contributions*: Thongchai Sooksawate, Kenta Kobayashi, and Kaoru Isa performed the experiments. Thongchai Sooksawate analyzed the data and prepared the figure.

The plasmid for NeuRet-MSCV-Cre vectors was obtained from a DNA fragment encoding the Cre recombinase gene (Kobayashi et al., 2004) and the murine stem cell virus promoter of pCL20c-MSCV (Hanawa et al., 2002, 2004). The pAAV-CAG-hChR2(H134R)-tdTomato and pAAV-EF1α-DIO-hChR2(E123T/T159C)-EYFP were provided from Dr. K. Svoboda and Dr. K. Deisseroth, respectively. The plasmid constructions are shown in Figure 1*A*.

### Viral vector preparation

NeuRet vectors were produced by the transfection of packaging plasmids (pCAGkGP4.1R and pCAG4-RTR2), envelope plasmid (pCAGGS-FuG-E), and transfer plasmid (pCL20-MSCV-Cre) into HEK293T cells. Cultured medium was collected and filtrated through a 0.45-μm Millex-HV filter unit (Millipore). Viral vector particles were pelleted by centrifugation and suspended in PBS. Suspended vector particles were purified by an ion-exchange column chromatography using Sepharose Q FF ion-exchange column (GE Healthcare) and AKTA prime plus chromatography apparatus (GE Healthcare). Collected fractions containing the vector particles were concentrated by ultrafiltration using a Vivaspin 10K MWCO filter (Vivascience). The copy number of RNA genome was estimated by a Lenti-X qRT-PCR titration kit (Clontech). Real-time quantitative PCR was performed in duplicate samples using the StepOne real-time PCR system (Applied Biosystems). AAV vectors were produced using the AAV Helper Free Expression System (Cell Biolabs). The packaging plasmids (pAAV-DJ and pHelper) and transfer plasmid (pAAV-EF1a-double floxed-hChR2(H134R)-EYFP-WPRE-HGHpA) were transfected into HEK293T cells. Harvested cells were lysed by repeated freezing and thawing, and a crude cell extract containing AAV vector particles was purified by ultracentrifugation with cesium chloride. After dialysis with PBS, vector particles were concentrated by ultrafiltration using an Amicon 10K MWCO filter (Merck Millipore). The copy number of viral genome (vg) was determined by the TaqMan Universal Master Mix II (Applied Biosystems).

### Viral vector injection

The entire time course of the experiments is shown in Figure 1*B*. We anesthetized the mice with an intraperitoneal injection of a mixture of ketamine (60 mg/kg body weight) and xylazine (10 mg/kg body weight). In addition, dexamethasone (5.5 mg/kg body weight) was injected intramuscularly as premedication. The head of the mouse was fixed to the stereotaxic apparatus (Narishige) and injections of the vectors were made from the dorsal approach. For the crossed (orienting) pathway neurons, NeuRet-MSCV-Cre (0.4–0.5 μl; titer, 1.1–30 × 10^11^ copies/ml) was injected into the medial PMRF on the left side using a thin glass micropipette (tip diameter, 50–70 μm) inclined 45° tip up in the sagittal plane (Franklin and Paxinos, 2008), −8.3 mm from the bregma, 0.7 mm lateral to the midline, and at 3.6 and 4.4 mm from the presumed dorsal surface of the cerebellar cortex. Injection site of NeuRet-MSCV-Cre is indicated in Figure 1*C*, left panel, lower insets, by a mock injection of Fluoro-Ruby (0.4 μl of 5% solution in 0.05 m PBS). Seven to 17 d after the NeuRet-MSCV-Cre injection, AAV-EF1α-DIO-hChR2(E123T/T159C)-EYFP (0.15–0.5 μl; titer, 4.5 × 10^10^ vg/μl) was injected into the SC on the right side (Fig. 1*C*, left). A small hole was made in the skull over the occipital cortex and a thin glass micropipette (tip diameter, 50–70 μm) was inserted vertically into the right SC, −3.8 to −4.0 mm from the bregma, 1.1–1.2 mm lateral to the midline, and at 1.1–1.6 and 1.4–2.3 mm from the presumed dorsal surface of the cerebral cortex (0.4–0.5 μl/point of injection). We used a syringe pump (ESP-32; Eicom, Legato 130; Muromachi Kikai Co) for the injection; the injection rate was 0.1 μl/min. Before removing the glass micropipette from the injection site, we waited for 5 min. For the uncrossed (defense) pathway neurons, NeuRet-MSCV-Cre (0.4–0.5 μl; titer, 1.1–30 × 10^11^ copies/ml) was injected into the right midbrain reticular formation (MRF) around the CnF using a thin glass micropipette inclined 45° tip up in the sagittal plane and aimed at −8.2–8.7 mm from bregma, 1.2 mm lateral to the midline. The injection site is indicated in Figure 1*C*, right panel, lower inset. Injection of AAV-EF1α-DIO-hChR2(E123T/T159C)-EYFP into the right SC was performed in the same way as the “crossed” SC-brainstem pathway group (Fig. 1*C*, right). For the control experiments, AAV-EF1α-DIO-EYFP, which lacks ChR2 was injected into the right SC to express only EYFP either in the crossed (orienting) or uncrossed (defense) pathway neurons.

We also prepared animals for optogenetic activation of the SC that was not selective for the crossed and ipsilateral pathways. For this non-selective activation of the SC, we injected AAV-CAG-hChR2(H134R)-tdTomato (1.9 × 10^9^ vg/μl) into the right SC using the approach described above in this section. We aimed to transfect all the cells in the SC, and then using the optogenetic activation of specific regions of the SC to investigate regional effects.

### Optic fiber implantation

Four to seven weeks after the viral injection(s), mice were anesthetized as mentioned above in the section “Viral vector injection”. Then, the 250- or 500-μm diameter plastic optical fiber (Lucir Inc.) was implanted through the cortex just above the SC surface using stereotaxic procedures. For the selective activation of output neurons of the SC, a 500-μm diameter optical fiber was implanted through the cortex just above the SC surface with the coordinate of −3.8 mm from bregma, 1.2 mm right of the midline, and 0.8–1.0 mm from the surface of the cerebral cortex. For activation of SC neurons in the non-selective paradigm, we divided the mice into three groups, caudo-lateral, rostro-medial, and central SC. A 500-μm optical fiber was implanted in the “central SC” group and a 250-μm optical fiber was implanted into “caudo-lateral” and “rostro-medial” groups. The stereotaxic coordinates for the caudo-lateral SC was −4.0 mm from bregma, 1.6 mm right of the midline and 0.8–1.0 mm from the surface of the cerebral cortex, that of the rostro-medial SC was −3.4 mm from bregma, 0.8 mm right of the midline and 0.8–1.0 mm from the surface of the cerebral cortex. For the central SC activation, the fiber was located at the same coordinates as of the selective pathway activation described above in this section. Because nearly the half of the SC is exposed outside the overlying cortex, the fiber track was just at the edge of the cortical tissue and the tip was above the surface of SC. Therefore, it was not possible for us to show the fiber track histologically.

### Behavioral effects of laser stimulation

Two to 7 d later, when the mice recovered from optical fiber implantation, the behavioral effects of the optical stimulation of the SC were investigated. To study the context dependency of the stimulus effects, the mice were placed either in a closed box (20 cm wide, 25 cm long, and 30 cm high) or on an open elevated circular field (open platform, 40-cm diameter and 1 m high from the floor) for testing the effect of blue illumination from a laser source (LUCIR, Model COME2-OFC-1, Lucir Inc.). The intensities for laser stimulation were 20–170 mW/mm^2^ for 250-μm diameter optical fiber and 36–260 mW/mm^2^ for the 500-μm diameter optical fiber. We stimulated the SC neurons with a single pulse of 50-, 100-, or 200-ms duration, or repetitive stimulation with 50 ms on–50 ms off duration at 10-Hz frequency with either 5×, 10×, or 20× repetitions. Behavioral data were analyzed with EthoVision XT 11.5 (Noldus). Stimulation with all of these parameters was repeated 10 times in each animal both in the closed box and on the open platform. Their sequences were randomized. Because adaptation of behavioral responses occurred as the stimulations were repeated, we did not stimulate >10 times with the same stimulation parameters. The number of mice used in each group was six to eight. In Results, we showed only the results of single pulse stimulation for 50 and 200 ms, and 20 times repetitive stimulation of 50-ms pulses for simplification. The results of other stimulation parameters fell in between these, and consequently are not shown to provide a simpler presentation.

**Figure 2.**
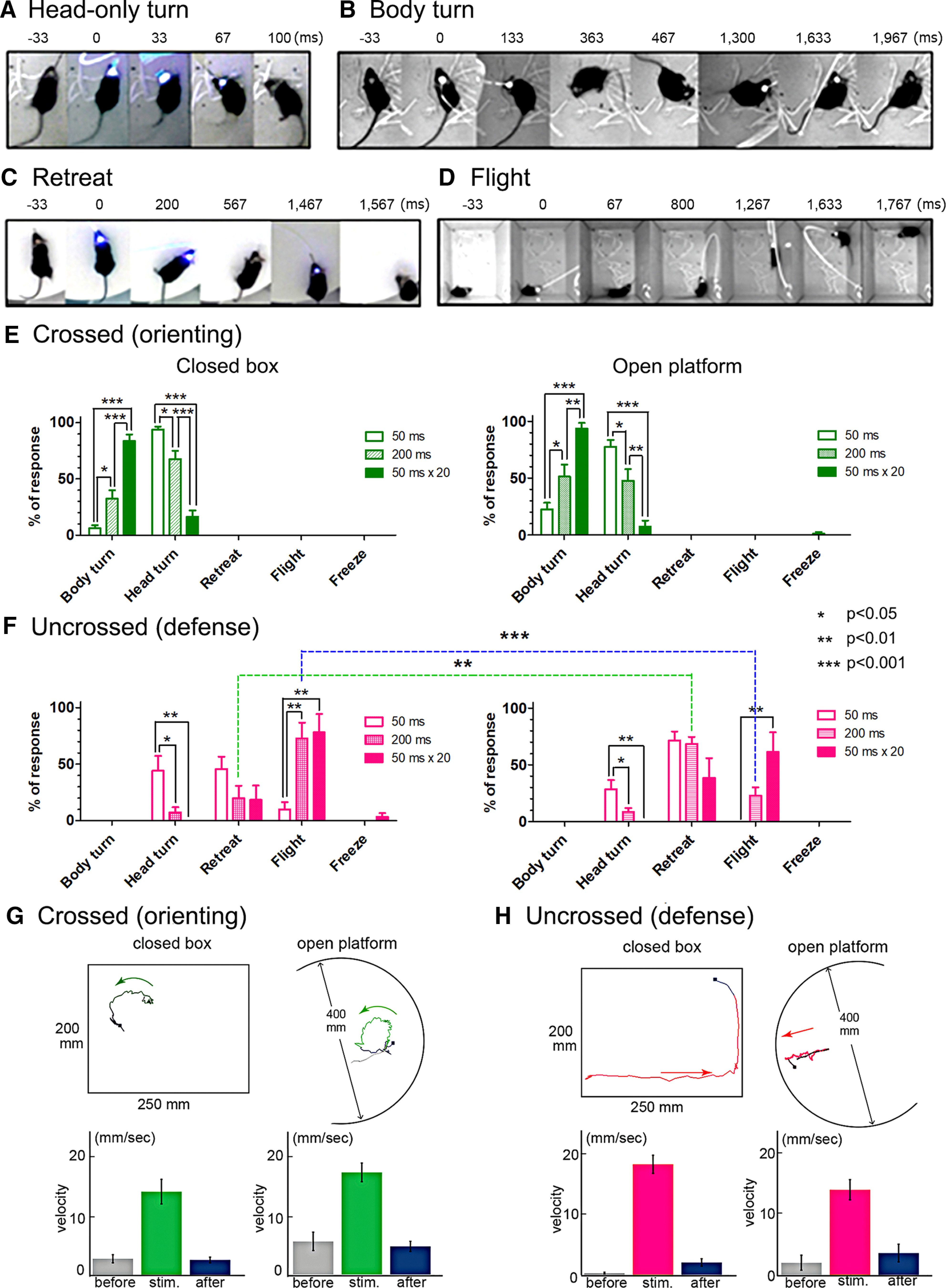
Categorization of behavioral responses to selective optogenetic activation of each SC output pathway. ***A–D***, Sequential photographs illustrating each of typical behavioral response patterns of the mice following optogenetic activation of SC neurons. ***E***, Categorization and percentage of responses in 10 trials of behavioral responses induced by the crossed (orienting) pathway with various stimulus intensities (50, 200, and 50 ms × 20). Left panel for the responses in the closed box and right panel for the responses on the open platform (*p < 0.05, **p < 0.01, ***p < 0.001, Bonferroni’s multiple comparison test, eight tested animals). ***F***, Categorization and percentage of responses in 10 trials of behavioral responses induced by the uncrossed SC-brainstem pathway with various stimulus intensities. Left panel for the responses in the closed box and right panel for the responses on the open platform (*p < 0.05, **p < 0.01, ***p < 0.001, Bonferroni’s multiple comparison test, seven tested animals). Frequencies of retreat and flight responses at 200-ms pulse were significantly different between the closed box and open platform environment (dotted green and blue lines with asterisks; **p < 0.01, ***p < 0.001, Bonferroni’s multiple comparison test, *n* = 7). ***G***, upper panel, Examples of the trajectory of the orienting responses of the mouse during stimulation of the crossed (orienting) pathway (20 trains of 50-ms pulses at 10 Hz) in the closed box and on the open platform. Lower panel, Histogram of the velocity of body movements before, during and after stimulation. ***H***, upper panel, Examples of the trajectory of the defense-like responses of the mouse during stimulation of the uncrossed (defense) pathway (20 trains of 50-ms pulses at 10 Hz) in the closed box and the open platform. Lower panel, Histogram of the velocity of body movements before, during and after stimulation. *Figure Contributions*: Thongchai Sooksawate, Kenta Kobayashi, and Kaoru Isa performed the experiments. Thongchai Sooksawate and Kaoru Isa analyzed the data and prepared the figure.

For the systematic evaluation of behavioral responses evoked by optical stimulation, they were classified as either orienting movements including head-only turn and contraversive body turn responses, or defense-like responses including retreat, flight and freezing responses (Fig. 2*A–D*). Defense-like responses often began with a quick upwardly directed head-only turn. This was often followed by backward movements (retreat) and/or fast forward run-away (flight). Note that head-only turns were directed in either ipsiversive or contraversive directions, or in the upward directions and observed for both crossed (orienting) and uncrossed (defense) pathway stimulation (especially at weaker stimulus intensities), and in both cases they were classified as “head-only turn.”

### *In vivo* electrophysiological recordings and electromyogram (EMG) recordings

After the behavioral experiments, 3–4 mice of each group were anesthetized with urethane (1.2–1.5 g/kg, i.p.) and the implanted optic fibers were removed. Dexamethasone (5.5 mg/kg) and atropine (0.1 mg/kg) were injected intramuscularly. Their heads were secured in the stereotaxic apparatus. A small patch of the skull was removed to expose the cortex overlying the SC. A 250-μm optical fiber was again lowered vertically into the brain with the same coordinates as the removed optical fiber. A thin tungsten electrode (2-MΩ resistance) was inclined by 20° and placed at 0.4 mm rostral to the rostral edge of optical fiber at the surface of the cortex and lowered into the right SC using a motor drive manipulator (DM System, Narishige). To confirm the location of the right SC, light flashes (Flash stimulator, SLS-3100, Nihon Kohden) were used to stimulate the left eye and monitor the light evoked potentials in the right SC. The depth of SCd was identified by observation of the reversal of visually evoked field potential (Katsuta and Isa, 2003), which was amplified by microelectrode amplifier (MEZ-8301, Nihon Kohden). Then, both eyes were covered by plastic tapes to avoid having the optic fiber light directly activate the retina. In this way, the effects of the cellular responses of the SCd cells to laser stimulation could be directly investigated. Data were digitized by analog-to-digital converter (Digidata 1440A, Molecular Devices) and acquired using a pClamp system (pClamp 10.2; Molecular Devices).

To investigate the shortest descending motor pathways to the neck muscles, EMG responses to the brief SC stimulation were measured. After behavioral testing, the mice were anesthetized with a ketamine-xylazine combination (60:10 mg/kg, i.p.). Dexamethasone (5.5 mg/kg) was injected intramuscularly. Then, the mouse head was secured in the stereotaxic apparatus. The EMG electrodes (stainless needles) were inserted into the dorsal neck muscles on both sides. The EMG responses from these muscles were measured during blue laser illumination. To clarify the contribution of reticulospinal neurons in the evoked EMG responses, a GABA_A_ receptor agonist, muscimol (0.1 μl, 0.1–1 mg/ml), was injected into the injection sites of NeuRet-MSCV-Cre in the left medial PMRF or right CnF using the same stereotaxic coordinates and method as the vector injections. The injections were intended to cover the wide area of both pontine and medullary reticular formation. As shown in the schematic diagram in Figure 5*A*, reticulospinal neurons mediating the inputs from the SC are known to possess both uncrossed and crossed connections to neck motoneurons (Isa and Sasaki, 2002). The number of mice used in each group was six to seven.

### Immunohistochemical assessments

At the end of the experiments, the mice were deeply anesthetized with an intraperitoneal injection of sodium pentobarbital (80 mg/kg body weight) and transcardially perfused with 0.05 m PBS followed by 4% paraformaldehyde in 0.1 m phosphate buffer (pH 7.4). The brain and spinal cord were cryoprotected and sectioned at a thickness of 40 μm using a sliding microtome (Retoratome REM-710, Yamato). The expression of ChR2-YFP in the double infected SC output neurons, including their somata and axons, were visualized either with direct fluorescence or anti-GFP immunohistochemistry. The latter were processed with Alexa Fluor 594 for fluorescence or with diaminobenzidine (DAB) as a chromagen and the DAB material was counterstained with neutral red. For fluorescence, the slices were dipped into the 0.3% Triton X-100 in PBS (PBST) containing 5% skimmed milk at room temperature and then incubated with a rabbit anti-GFP antibody (1:2000; Life Technologies) in PBST for 16 h at 4°C. The sections were washed in PBST and incubated in Alexa Fluor 594-conjugated anti-rabbit IgG (1:200; Life Technologies) in PBST. For the DAB staining, the sections were rinsed and incubated in the blocking solution of 0.6% H_2_O_2_ in Dent’s fixative followed by being rinsed and incubated in PBST containing 5% skimmed milk and in continued first antibody as mentioned above in this section. Then they were washed in PBST and incubated in biotinylated goat anti-rabbit IgG (1:200; Vector Laboratories) followed by reaction in A and B solutions of ABC-Elite (1:200; Vector laboratories). The label was visualized with DAB (1:10,000; Wako) containing 1% Nickel ammonium sulfate and 0.0003% H_2_O_2_ in Tris-buffered saline. Images of the labeled neurons and fibers with fluorescence or DAB were taken using light microscope (BZ-9000, BZ-X710, Keyence) and the drawings were made using a light microscope equipped with a camera lucida (BX-51, Olympus). A fluorescent photomicrograph of the SC was taken using fluorescent microscope (Akioplan 2, Zeiss).

### Statistical analysis

Data are expressed as mean ± SEM. Significance was tested by Student’s unpaired *t* test or one-way ANOVA and Bonferroni’s multiple comparison test where applicable, and *p* < 0.05 was considered to be significant.

## Results

### Pathway-selective optogenetic activation of SC output neurons

For selective activation of the descending pathway from the SC projecting to the contralateral medial PMRF, one group of mice was injected with the retrograde gene transfer vector, NeuRet-MSCV-Cre (Fig. 1*A2*) into the left PMRF (Fig. 1*C*, left panel, lower inset) and AAV-DJ-EF1α-DIO-hChR2(E123T/T159C)-EYFP (Fig. 1*A1*) into the right (contralateral) SC (Fig. 1*C*, left). In these mice, ChR2 was expressed exclusively in SC neurons projecting to the crossed (orienting) pathway. In the second group of mice, the former vector was injected into the right MRF around the CnF (Fig. 1*C*, right panel, lower inset) and the second vector was injected into the right (ipsilateral) SC (Fig. 1*C*, right). In these animals, the expression of ChR2 was restricted to the neurons whose axons projected to the uncrossed (defense) pathway. As shown in Figure 1*C*, left, crossed (orienting) pathway neurons are multipolar medium-large sized neurons, while soma size of the uncrossed (defense) pathway neurons (Fig. 1*C*, right) tended to be smaller than the crossed (orienting) pathway neurons. After the behavioral testing, electrophysiological experiments were conducted under anesthesia (Fig. 1*B*). Electrophysiological recording from SC neurons in both groups showed that when optical stimulation with blue laser (473 nm wavelength) was applied to the right SC, both the crossed (orienting) and uncrossed (defense) pathway neurons were robustly activated (Fig. 1*D*). The latencies of the spiking responses after the laser onset were 8.01 ± 0.66 ms (*n* = 23) for the crossed (orienting) pathway neurons and 9.27 ± 1.54 ms (*n* = 13) for the uncrossed (defense) pathway neurons, respectively. Following repetitive optical stimulation (10–20 trains of 50-ms pulses at 10 Hz) both types of SC neurons were reliably activated.

To investigate the extent to which behavioral responses were context dependent, observations were made either in a small box (25 × 20 cm), to which animals had been habituated for 1 h before testing, or on a large open platform (an elevated circle, 40 cm in diameter). Mice generally feel more secure in the enclosed space, where they can use the corner of walls for protection from predators, so that these two testing environments provide different behavioral contexts to the animals. Sequences of presentation of optical stimulation were randomized among individual animals.

Figure 2*E*, left and right panels, shows the behavioral responses induced by the optical stimulation of the crossed (orienting) pathway neurons with a large optic fiber (500 μm in diameter) located centrally on the surface of the SC in the closed box and on the open platform, respectively (eight tested animals). In the closed box, a brief stimulation (single pulses 50 ms) induced contraversive horizontal head-only turns (Fig. 2*A*; demonstrated in Movie 1) on average in 94% of the trials in the closed box (Fig. 2*E*, left panel). Progressively extended stimulations to 200 ms and 20 trains of 50-ms pulses at 10 Hz significantly increased the frequency of body turns (Fig. 2*B*; demonstrated in Movie 2) and reduced the head-only turns (*p* < 0.05 or *p* < 0.001, Bonferroni’s multiple comparison test; Fig. 2*E*, left panel). With single pulse 200-ms stimulation, 68% of the responses were head-only turns, and body turn responses were induced in 32% of the trials. When the stimulus duration was increased from single 50-ms pulses to 20 trains of 50-ms pulses at 10 Hz, frequency of head-only turns decreased to 16% and that of body turns increased to 84% in the closed box (Fig. 2*E*, left panel). All optically evoked movements were elicited at short latency (<30 ms), almost always appearing in the first video frame following the laser onset. When the same stimulus was applied on the open platform, single 50-ms pulses induced head-only turn responses in 78% of the trials (Fig. 2*E*, right panel). With single pulse 200-ms stimulation, 48% of the responses were head-only turns, and body turn responses were induced in 51% of the trials. When the stimulus duration was increased from single 50-ms pulses to 20 trains at 10 Hz, frequency of head-only turns decreased to 6%, and frequency of body turns significantly increased to 94% (*p* < 0.001, Bonferroni’s multiple comparison test; Fig. 2*E*, right panel; demonstrated in Movie 3). No significant difference between the percentages of responses could be found for both the brief stimulation and the extended stimulation between the two environments (Bonferroni’s multiple comparison test). Velocities of the circling responses in the closed box and open platform were not significantly different (*p* > 0.05, Student’s *t* test; Fig. 2*G*). Thus, similar contraversive responses were evoked in both testing environments, and these movements were considered to be context independent. As a control, we confirmed that laser stimulation of the SC with red color (635 nm, 163 mW/mm^2^) did not induce any behavior change, head turn, body turn or defensive response in three mice with ChR2 expression in the crossed pathway. In addition, we also stimulated the SC in the mice with injection of a viral vector lacking ChR2 sequence (*n* = 3) and confirmed that no movement was elicited.

Movie 1. Head-only turn response. Pathway selective optogenetic activation-induced head-only turn of mouse in the crossed (orienting) pathway group to blue laser (50-ms duration) in the closed box. 10.1523/ENEURO.0271-20.2020.video.1

Movie 2. Body turn in closed box. Pathway selective optogenetic activation-induced body turn response of mouse in the crossed (orienting) pathway group to blue laser (50-ms duration, 10 Hz, 20 trains) in the closed box. 10.1523/ENEURO.0271-20.2020.video.2

Movie 3. Body turn response in open platform. Pathway selective optogenetic activation-induced body turn response of mouse in the crossed (orienting) pathway group to blue laser (50-ms duration, 10 Hz, 20 trains) on the open platform. This mouse was the same one as in Movie 2. 10.1523/ENEURO.0271-20.2020.video.3

Figure 2*F*, left and right panels, shows the behavioral responses induced by the optical stimulation of the uncrossed (defense) pathway neurons with a large optic fiber (500 μm in diameter) located centrally on the surface of the SC in the closed box and on the open platform, respectively (seven tested animals). In the closed box, a brief optical stimulation (single 50-ms pulses) induced head-only turns in 44% and retreat responses in 46% of the trials on average (*n* = 7; Fig. 2*F*, left panel). It should be noted that a small, upward head-only turn was present in the first video frame of the retreat response trials. With single pulse 200-ms stimulation, 73% of the responses were flight, and retreat responses were induced in 20% of the trials. With extended stimulus duration (20 trains of 50-ms pulses at 10 Hz), the frequency of head-only turn responses became zero, while frequency of retreat decreased to 16% and flight responses significantly increased to 81% (*p* < 0.01, Bonferroni’s multiple comparison test; Fig. 2*F*, left panel). The flight responses were directed mainly forward or sideways, not purely backwards, and the animals appeared to be hiding in the corner. There was no fixed trend in direction of initial flight responses. An important novel finding was that the defense-like responses elicited by activation of the uncrossed (defense) pathways were context-dependent, as described below. As demonstrated in the Movies 4, 5, the same optical stimulation parameter in the closed box frequently induced flight responses mainly consisting of moving forward from corner to corner (Movie 4), while on the open platform retreat was the primary response in the same animal (Movie 5). In the closed box, because the uncrossed pathway was stimulated only on the right side, the animals tended to move to the right-forward or right-lateral direction regardless of the distance from the wall. After coming close to the wall, they turned and ran in parallel to the wall. On the open platform, with the single 50-ms pulse, oblique-upward head-only turns were induced in 29% of the trials and retreat was induced in 71% of the trials (*n* = 7; Fig. 2*F*, right panel). The frequency of retreat responses on the open platform (71%) was higher than in the closed box (46%). The same tendency could be more clearly observed for the extended stimulus. With single pulse 200-ms stimulation on the open platform, flight responses appeared in 23% of the trials, while retreat responses were induced in 69% of the trials. The frequency of retreat responses on the open platform (69%) was significantly higher than in the closed box (20%; *p* < 0.01, Bonferroni’s multiple comparison test; Fig. 2*F*, horizontal green dotted line across left and right panels). In contrast, the frequency of flight responses on the open platform (23%) was significantly lower than in the closed box (73%; *p* < 0.001, Bonferroni’s multiple comparison test; Fig. 2*F*, horizontal blue dotted line across left and right panels). On the open platform, with extended stimulus duration (20 trains of 50-ms pulses at 10 Hz), the frequency of head-only turn responses became zero, and the frequency of retreat also decreased to 39%, and the frequency of flight responses increased to 61%. The velocities of flight responses in the closed box were significantly faster than those of retreat responses in the open platform (*p* < 0.05, Student’s *t* test; Fig. 2*H*). In summary, selective activation of the uncrossed (defense) pathway was associated with active defense-like movements, such as retreat or flight responses, and induction of these responses depended on the environment where the animals were placed (context-dependent). Here, we have to point out that passive freezing responses, another type of defense-like behaviors, were rarely observed (in case of 20 trains of 50-ms pulses, freezing response was observed in only 3% in the closed box and 0% in the open platform, *n* = 7). This result will be discussed later. As a control, we also stimulated the SC in the mice with injection of a viral vector lacking ChR2 (*n* = 3) and confirmed that no movements were elicited.

Movie 4. Flight response in closed box. Pathway selective optogenetic activation-induced flight response of mouse in the uncrossed (defense) pathway group to blue laser (50-ms duration, 10 Hz, 20 trains) in the closed box. 10.1523/ENEURO.0271-20.2020.video.4

Movie 5. Retreat response in open platform. Pathway selective optogenetic activation-induced retreat response of mouse in the uncrossed (defense) pathway group to blue laser (50-ms duration, 10 Hz, 20 trains) on the open platform. 10.1523/ENEURO.0271-20.2020.video.5

### Axonal trajectories of neurons consisting the crossed (orienting) and uncrossed (defense) pathways

**Figure 3.**
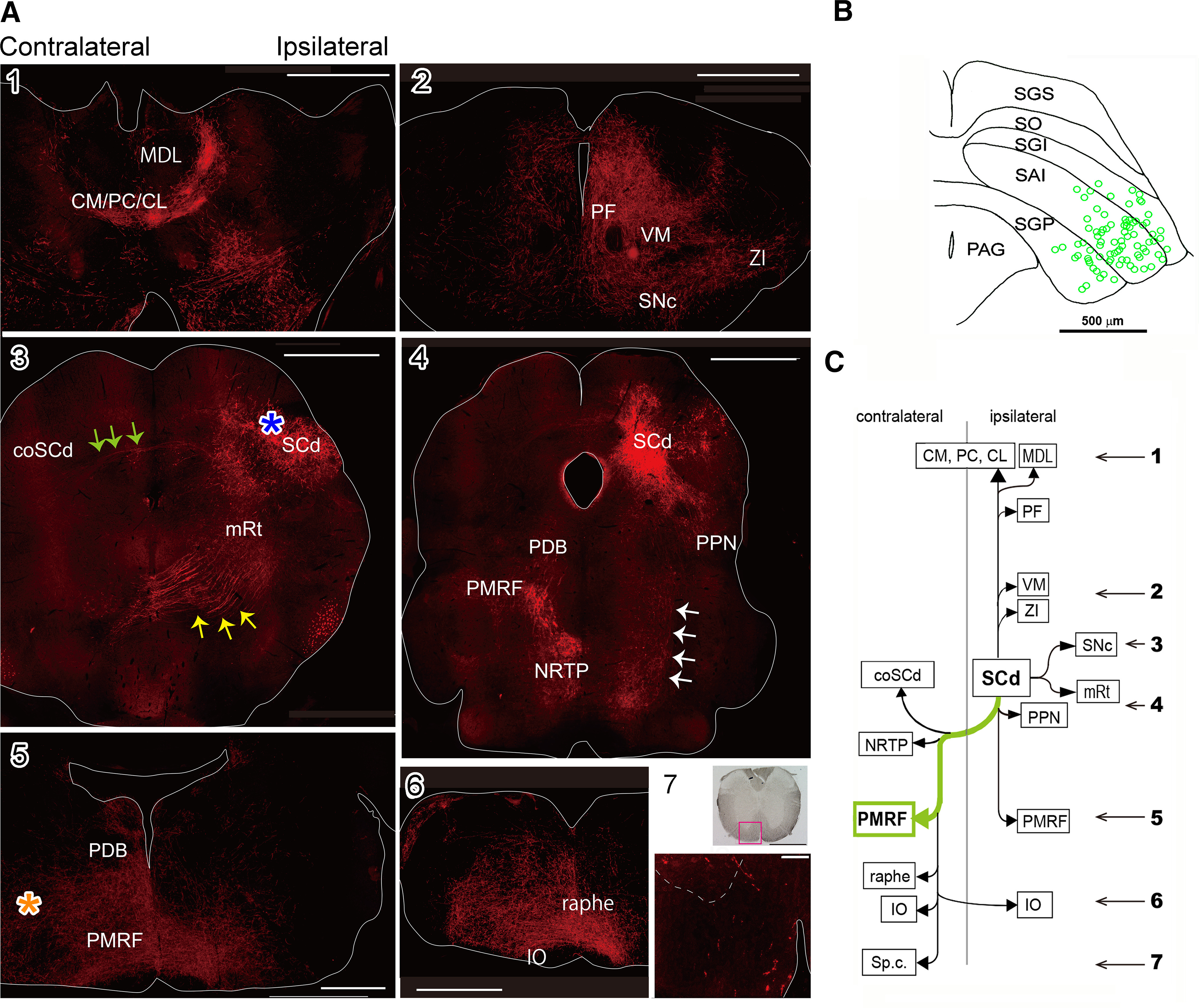
Axonal trajectories of the crossed (orienting) pathway neurons visualized by anti-GFP immunohistochemistry with Alexa Fluor 594. ***A***, Crossed (orienting) pathway. ***1–6***, Photomicrographs of the frontal sections of the diencephalon, brainstem. Scale bars = 1 mm. ***7***, Spinal cord (C1 level). Upper panel, Low-magnification bright field view (scale bar = 1 mm). Lower panel, High-magnification fluorescent view of the square area in the upper panel (scale bar = 100 μm). ***B***, A diagram showing the location of the somata of crossed (orienting) pathway neurons. ***C***, Targets of the crossed (orienting) pathway neurons. The numerals with arrows indicate the rostrocaudal levels of photomicrographs in ***A1–A7***. An asterisk indicates the injection site of viral vector in PMRF. CM/PC/CL: rostral intralaminar thalamic nuclei, such as central medial thalamic nucleus (CM)/paracentral thalamic nucleus (PC)/centrolateral thalamic nucleus (CL), coSCd; contralateral SC deeper layers, IO: inferior olive, MDL: mediodorsal thalamic nucleus lateral part, mRt: mesencephalic reticular formation, NRTP: nucleus reticularis tegmenti pontis, PDB: predorsal bundle, PF: parafascicular thalamic nucleus, PMRF: medial PMRF, PPN: pedunculopontine nucleus, SAI: intermediate white layer, SC: superior colliculus, SCd: SC deeper layers, SGP: deep gray layer, SGS: superficial gray layer, SNc: substantia nigra pars compacta, SO: optic layer, Sp.c.: spinal cord, VM: ventral mediothalamic nucleus, ZI: zona incerta. *Figure Contributions*: Thongchai Sooksawate, Kenta Kobayashi, and Kaoru Isa performed the experiments. Kaoru Isa, Peter Redgrave, and Tadashi Isa analyzed the data.

**Figure 4.**
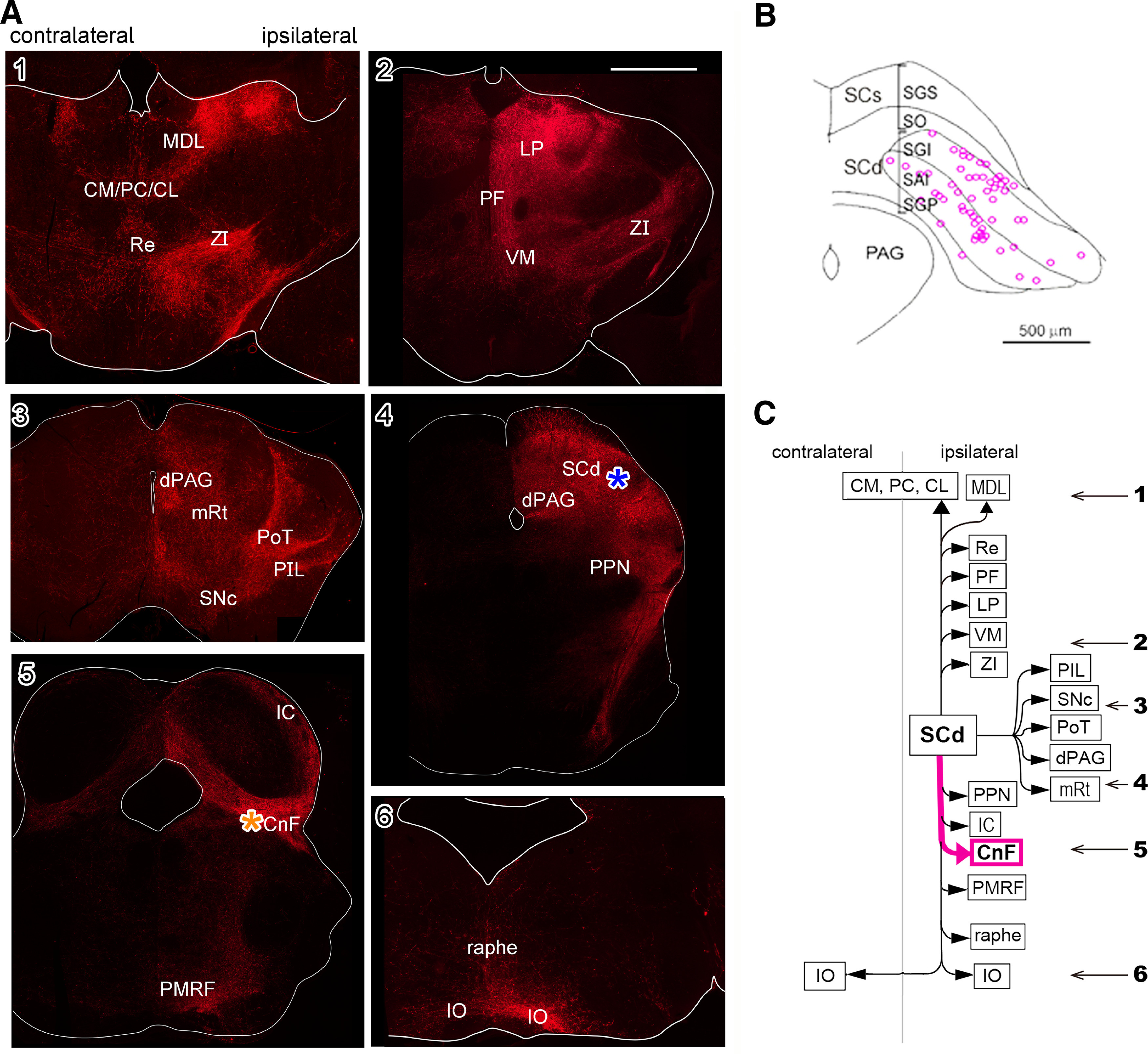
Axonal trajectories of the uncrossed (defense) pathway neurons visualized by anti-GFP immunohistochemistry with Alexa Fluor 594. ***A***, Uncrossed (defense) pathway. ***1–6***, Photomicrographs of the frontal sections of the diencephalon and brainstem. Scale bars = 1 mm. ***B***, A diagram showing location of the somata of uncrossed (defense) pathway neurons. ***C***, Targets of the uncrossed (defense) pathway neurons. The numerals with arrows indicate the rostrocaudal levels of photomicrographs in ***A1–A6***. An asterisk indicates the injection site of viral vector in the right MRF around CnF. The same abbreviations as Figure 3 and additional abbreviations: dPAG: dorsal periaqueductal gray matter, IC: inferior colliculus, LP: lateral posterior thalamic nucleus, PIL: posterior intralaminar nucleus, PoT: posterior thalamic nucleus, triangular, Re: reuniens thalamic nucleus. *Figure Contributions*: Thongchai Sooksawate, Kenta Kobayashi, and Kaoru Isa performed the experiments. Kaoru Isa, Peter Redgrave, and Tadashi Isa analyzed the data.

After the mice were killed, morphology of the activated cells was visualized with anti-GFP immunohistochemistry with Alexa Fluor 594 (Figs. 3, 4). The somata and axonal arbor of the crossed (orienting) pathway were labeled by an injection into the left PMRF (for approximate location, see Fig. 3*A5*, asterisk). The somata of the cells of origin of this pathway were concentrated in the most lateral portion of the SCd (Fig. 3*B*). Some fibers crossed the midline in the tectal commissure (Fig. 3*A3*, green arrows) and terminated in the most lateral portion of the contralateral SCd, the mirrored location of the cells of origin (Fig. 3*A3*, coSCd). A majority of axons of these neurons crossed the midline in the ventral tegmental decussation (Fig. 3*A3*, yellow arrows) and turned caudally to join the contralateral PDB (Fig. 3*A4*,*A5*). The axons continued into the medial PMRF, where they approached the injection sites of the retrograde vector NeuRet-MSCV-Cre, and continued to the nucleus reticularis tegmenti pontis (NRTP), PMRF, raphe nuclei (raphe), inferior olive (IO), and spinal cord (Sp.c.) on the contralateral side (Fig. 3*A4-A7*). On the ipsilateral side, some labeled axons descended to the ipsilateral pedunculopontine nucleus (PPN) and to the most ventral aspect of ipsilateral PMRF and to the IO (Fig. 3*A4*, white arrows, *A5*,*A6*). In addition, the ascending collaterals could be traced to the mediodorsal thalamic nucleus lateral part (MDL) and rostral intralaminar thalamic nuclei such as paracentral thalamic nucleus (PC) and centrolateral thalamic nucleus (CL) on the ipsilateral side, and central medial thalamic nucleus (CM) on both sides (Fig. 3*A1*). The ascending axons also projected to the ipsilateral mesencephalic reticular formation (mRt), parafascicular thalamic nucleus (PF), ventral mediothalamic nucleus (VM), substantia nigra pars compacta (SNc), and zona incerta (ZI; Fig. 3*A2*,*A3*). The target areas of the crossed (orienting) pathway neurons are summarized in Figure 3*C*. Thus, it was turned out that the classical crossed descending pathway has some ipsilaterally descending collaterals.

In contrast, although widely located throughout the rostral-caudal axis of the SCd, the somata of the neurons consisting the uncrossed (defense) pathway labeled by an injection into the right MRF around the CnF (for approximate location, see Fig. 4*A5*, asterisk) were located more medially (Fig. 4*B*) than those of the crossed (defense) pathway neurons (Fig. 3*B*). The descending axons projected to the inferior colliculus (IC), CnF, PPN, and PMRF on the ipsilateral side, and bilaterally to the IO (Fig. 4*A4-A6*). At the level of the SC, the axons projected to the SNc, posterior thalamic nucleus triangular (PoT), dorsal periaqueductal gray matter (dPAG) and laterally in the mesencephalic reticular formation (mRt; Fig. 4*A3*). The ascending axons of the uncrossed (defense) neurons could be traced to the ipsilateral CM, PC, CL, MDL, PF, reuniens thalamic nucleus (Re), lateral posterior thalamic nucleus (LP), VM, ZI, and posterior intralaminar thalamic nucleus (PIL; Fig. 4*A1-A3*). The target areas of the uncrossed (defense) pathway neurons are summarized in Figure 4*C*.

These results not only confirmed the previously described connectivity patterns of the crossed and uncrossed descending projection of the SC (Redgrave et al., 1987), but also revealed some novel projections. For example, the ipsilaterally descending branches of crossed (orienting) pathway neurons targeting ipsilateral SNc, PPN, and ventral part of PMRF (Fig. 3*A4*, white arrows). They also terminate in the ipsilateral IO (Fig. 3*A6*). Moreover, it was turned out that the classical uncrossed descending pathway has some collateral projection to the contralateral IO (Fig. 4*A6*).

All these tracing studies revealed more fine structures of the both systems. Hereafter, we rename the classical crossed descending pathway as “tectal orienting pathway” and the classical “uncrossed” descending pathway as “tectal defense pathway.”

### EMG responses of dorsal neck muscles following optical stimulation of the tectal orienting and tectal defense pathways

**Figure 5.**
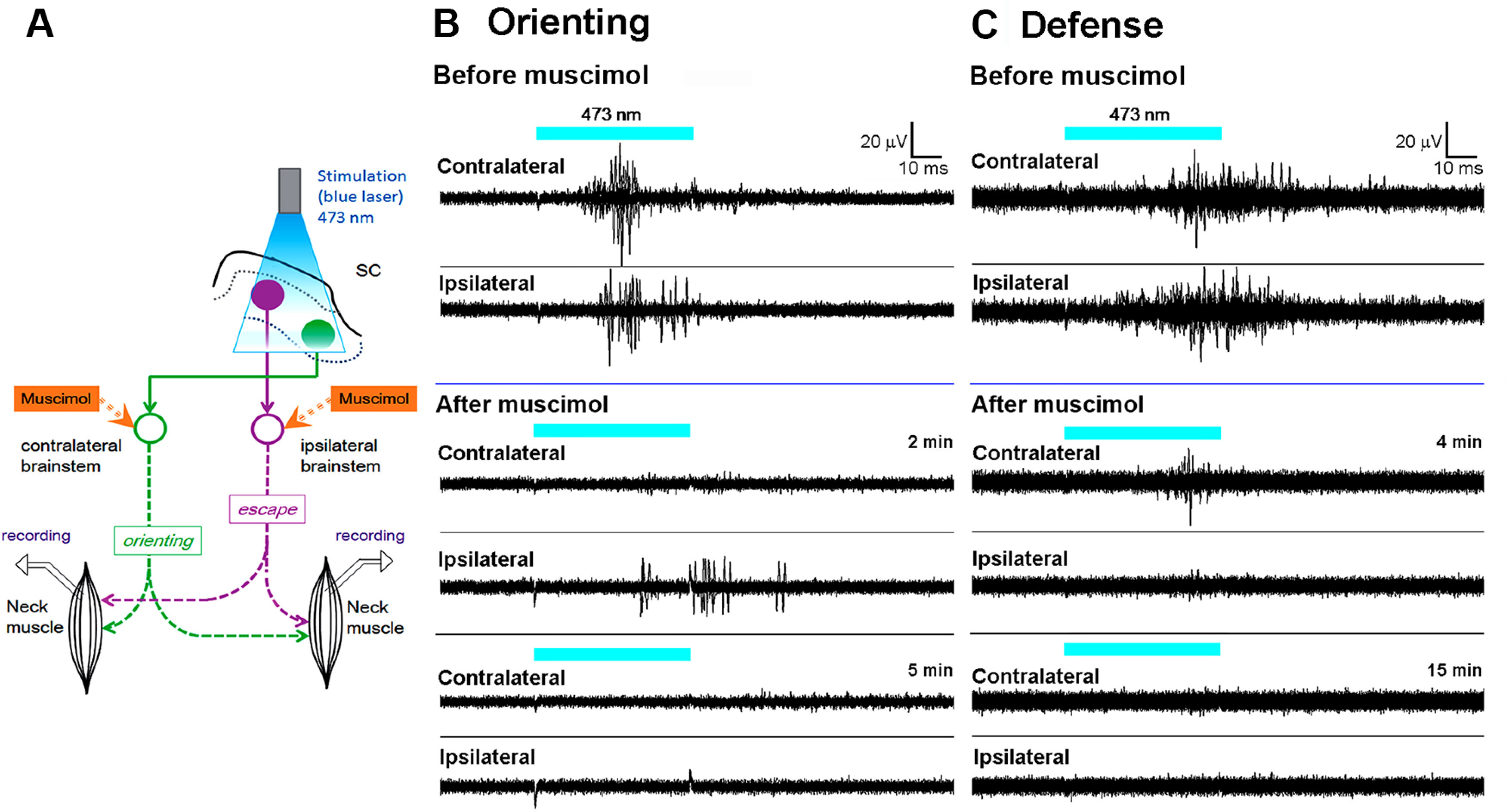
EMG responses of dorsal neck muscles to optogenetic stimulation of the SC neurons. ***A***, Schematic diagram showing the experimental protocol. ***B***, EMG response to the activation of the tectal orienting pathway, which was inhibited by a muscimol at 2 and 5 min after injection into the brainstem. ***C***, EMG responses to the activation of the tectal defense pathway, which were also abolished by unilateral microinjection of muscimol at 2 and 5 min after injection into the brainstem. The horizontal blue lines above individual traces indicate the period of laser irradiation. *Figure Contributions*: Thongchai Sooksawate, Kenta Kobayashi, and Kaoru Isa performed the experiments. Thongchai Sooksawate analyzed the data. Thongchai Sooksawate and Kaoru Isa prepared the figure.

To further investigate the synaptic connectivity between the tectal orienting and defense pathways and spinal motoneurons responsible for head turns, we recorded the EMG responses of dorsal neck muscles evoked by ipsilateral or contralateral stimulation of the SC in anaesthetized animals (Fig. 5*A*). When the tectal orienting pathway was activated, the mean latency of evoked EMG responses in contralateral (left) muscles was 12.85 ± 0.96 ms (*n* = 6), and in ipsilateral (right) muscles was 13.01 ± 1.17 ms (*n* = 6; Fig. 5*B*). Considering the previous observation that spiking activity in tectal orienting pathway neurons was induced at the latency of 8.01 ± 0.66 ms (*n* = 23) after the laser onset (Fig. 1*D1*), the conduction time from the SC to the neck muscles must have been in the range of 4–5 ms, on both sides. These ultra-short latency muscular responses were completely abolished by microinjections of 0.1 μl muscimol (0.1–1.0 mg/ml) into the left PMRF (Fig. 5*B*), the injection sites of NeuRet-MSCV-Cre (Fig. 3*A5*, asterisk). This result suggests that activation of the dorsal neck muscles by the SC is mediated primarily by tecto-reticulo-spinal pathways with relay neurons in the PMRF. They have both uncrossed or crossed connections to neck motoneurons (Isa and Sasaki, 2002). Following activation of the tectal defense pathway (Fig. 5*C*), the mean latency of EMG responses in the contralateral (left) muscles was 13.52 ± 1.04 ms (*n* = 7), and in the ipsilateral (right) muscles was 12.41 ± 1.13 ms (*n* = 7). Considering again that the average latencies of optically evoked spiking responses of the relevant SCd neurons was 9.27 ± 1.54 ms (*n* = 13; Fig. 1*D2*), the conduction time in the uncrossed descending projection from the SC to the neck muscles was ∼3–4 ms. Again, these short latency EMG responses were abolished after microinjections of muscimol into the brainstem close to the right CnF (Fig. 5*C*), the injection site of retrograde vector (Fig. 4*A4*, asterisk). These results suggest that the excitatory drive from the SC to the dorsal neck muscles is primarily mediated via the tectal defense pathway with a critical relay in the brainstem reticular formation around the CnF. These may include nearby reticulospinal neurons with crossed or uncrossed descending projections to the neck motoneurons (Fig. 5*A*, schematic diagram; Isa and Sasaki, 2002). Taken together, the short latencies of the evoked EMG responses may be explained by the synaptic connectivity from the SC to neck muscles via both tectal orienting and defense pathways are di- or oligosynaptic and mediated by the crossed and uncrossed reticulospinal pathways.

### Nonselective optogenetic activation of the SC neurons

**Figure 6.**
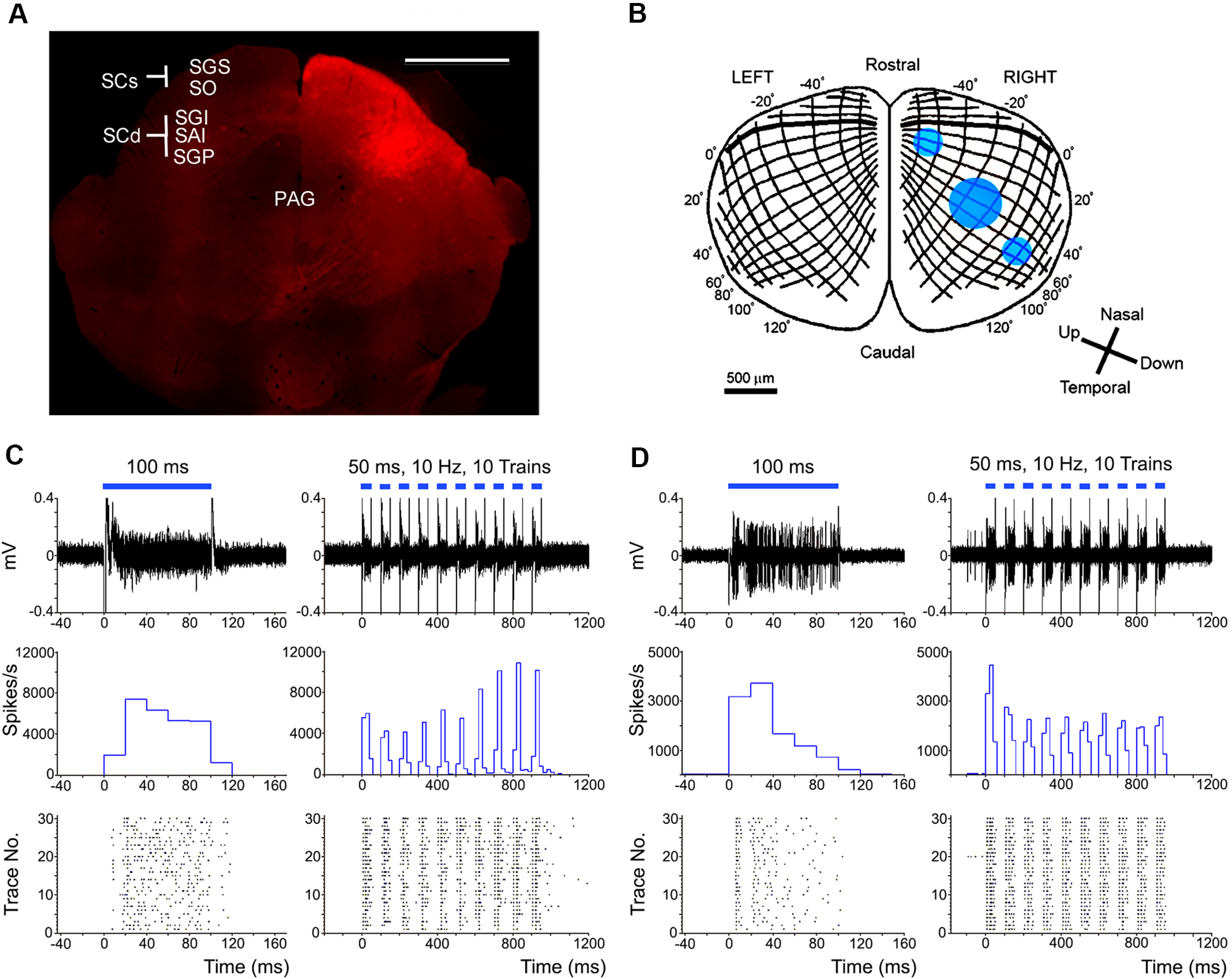
Nonselective optogenetic activation of the SC neurons. ***A***, Fluorescent photomicrographs of the SC that expressed ChR2 and tdTomato. Scale bar = 1 mm. ***B***, Site of laser stimulation on the topographic map of the mouse SC. ***C***, Responses of mouse SCs neuron to blue laser stimulation; raw traces (upper row), peristimulus time histogram (PSTH; middle row), and raster plots (lower row). ***D***, Responses of mouse SCd neuron to blue laser stimulation; raw traces (upper row), PSTH (middle row), and raster plots (lower row). The horizontal blue lines above individual traces indicate the period of laser irradiation; single, long pulse (100 ms) on the left (horizontal blue line), and repetitive stimulation with short pulses on the right (10 trains of 50-ms duration pulses with 50-ms interval (10 Hz), horizontal blue broken line). PAG: periaqueductal gray matter, SAI: intermediate white layer, SC: superior colliculus, SCd: SC deeper layers, SCs: SC superficial layers, SGI: intermediate gray layer, SGP: deep gray layer, SGS: superficial gray layer, SO: optic layer. *Figure Contributions*: Thongchai Sooksawate, Kenta Kobayashi, and Kaoru Isa performed the experiments. Thongchai Sooksawate analyzed the data. Thongchai Sooksawate and Kaoru Isa prepared the figure.

To compare the effects of pathway-selective activation with those of nonselective activation of the SC, AAV-CAG-Ch2R(H134R)-tdTomato was injected into the SC. As shown in Figure 6*A*, neurons in virtually the whole SC, including both SCs and SCd, expressed ChR2-tdTomato.Optical stimulation with a blue laser was applied at 3 locations (Fig. 6*B*) in SC; (1) a 250-μm diameter fiber was used to stimulate neurons representing the lower visual field in the caudo-lateral SC, (2) a 250-μm diameter fiber was also used to activate the rostro-medial aspect of the SC representing the upper visual field, and (3) a larger 500-μm diameter fiber was placed in the central part to activate a wide area of the SC. Under anesthesia, the neurons in the SCs and SCd located below the optic fiber were reliably activated by the laser stimulation, in response to both prolonged pulses of laser stimulation (100-ms duration) and repetitive trains of short pulses (10 trains of 50-ms pulse at 10 Hz; Fig. 6*C*,*D*, respectively).

**Figure 7.**
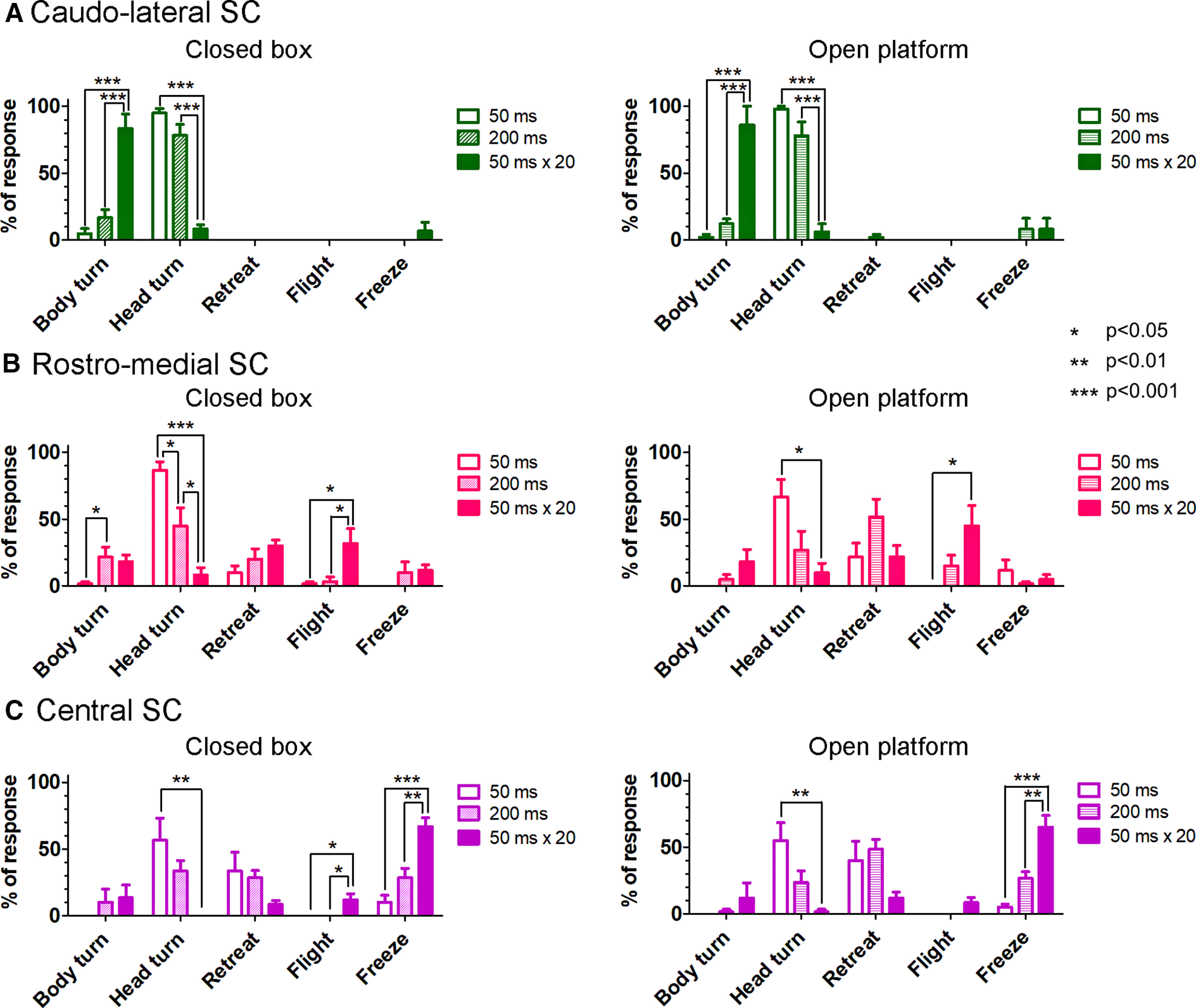
Categorization of behavioral responses to nonselective optogenetic activation of the SC neurons in the closed box or on the open platform environment. Behavioral response categories (body turn, head-only turn, retreat, flight, and freezing responses) are plotted on the horizontal axis and their percentage of occurrence in 10 trials to stimulation with each parameter on the vertical axis. ***A***, Stimulation of the caudo-lateral SC by a 250-μm diameter fiber with various stimulation parameters (50, 200, and 50 ms × 20). Left panel for the responses in the closed box and right panel for the responses on the open platform (*p < 0.05, **p < 0.01, ***p < 0.001, Bonferroni’s multiple comparison test, six tested animals). ***B***, Stimulation of the rostro-medial SC by a 250-μm diameter fiber with various stimulation parameters. The same arrangement as ***A*** (six tested animals) ***C***, Stimulation of the central SC by a 500-μm diameter fiber with various stimulation parameters. The same arrangement as ***A***, ***B*** (six tested animals). *Figure Contributions*: Thongchai Sooksawate, Kenta Kobayashi, and Kaoru Isa performed the experiments. Thongchai Sooksawate and Kaoru Isa analyzed the data and prepared the figure.

Figure 7*A*, left and right, show the behavioral responses induced by the optical stimulation of the caudo-lateral SC by a 250-μm diameter fiber in the closed box and on the open platform, respectively (six tested animals). A brief optical stimulation (single 50-ms pulses) induced contraversive orienting responses of the head (head-only turn) in the awake condition [95% in closed box (Fig. 7*A* , left panel) and 98% in open platform (Fig. 7*A* , right panel)]. With the extended stimulation, large angular rotations of the head eventually followed by contraversive body turn were observed. When the stimulus duration was increased from single 50-ms pulses to single 200-ms pulses and 20 trains of 50-ms pulses at 10 Hz, the frequency of body turn significantly increased from 5% to 17% and 83%, and then that of head-only turn significantly decreased from 95% to 78% and 8%, respectively (*p* < 0.001, Bonferroni’s multiple comparison test), in the closed box. On the open platform, the same stimulus parameters also significantly increased the frequency of body turn from 2% to 12% and 86%, and significantly decreased that of head-only turn from 98% to 78% and 6%, respectively (*p* < 0.001, Bonferroni’s multiple comparison test; Fig. 7*A*, right panel). No significant difference between the percentages of responses for each stimulus parameter between the two environments could be found for either the brief stimulation or the extended stimulation (Bonferroni’s multiple comparison test). Thus, the evoked responses were context independent.

Figure 7*B*, left and right, shows the behavioral responses induced by the optical stimulation of the rostro-medial SC by a 250-μm diameter fiber in the closed box and on the open platform, respectively (six tested animals). In the closed box, a brief stimulation (single 50-ms pulses) tended to induce mainly an upward and slightly ipsiversive head-only turn (87%). When the pulse width was increased (single 200-ms pulses), the frequencies we observed were 45% for head only turn, 22% for body turn, 20% for retreat, 3% for flight and 10% for freezing (Fig. 7*B*, left panel). On the open platform, they were 5%, 27%, 52%, 15%, and 2%, respectively (Fig. 7*B*, right panel). Thus, various responses were induced and there was no statistical difference between the closed box and open platform (Bonferroni’s multiple comparison test). In case of extended stimulation (20 trains of 50-ms pulses at 10 Hz), frequency of flight responses tended to increase [to 32% in the closed box (Fig. 7*B*, left panel) and 45% in the open platform (Fig. 7*B*, right panel, *p* < 0.05, Bonferroni’s multiple comparison test)] and again, there was no statistical difference between the closed box and open platform (Bonferroni’s multiple comparison test). We confirmed that laser stimulation of the rostro-medial SC with red color (635 nm, 163 mW/mm^2^) did not induce any behavior change, head turn, body turn, or defensive response in three mice as a control.

Figure 7*C*, left and right panels, shows the behavioral responses induced by the optical stimulation of the wide area of the SC by the 500-μm diameter optic fiber placed over the central SC in the closed box and on the open platform, respectively (six tested animals). A brief stimulation (50 ms) evoked head-only turn [57% in closed box (Fig. 7*C*, left panel) and 55% in open platform (Fig. 7*C*, right panel)]. With the increases in the duration of optical stimulation (20 trains of 50-ms pulses at 10 Hz), the frequency of freezing responses (exemplified in Movie 6, defined as movement halt during and for a few seconds after the stimulus offset) markedly increased on average, to 67% in the closed box (*p* < 0.01 and *p* < 0.001, Bonferroni’s multiple comparison test; Fig. 7*C*, left panel) and 65% on the open platform (*p* < 0.01 and *p* < 0.001, Bonferroni’s multiple comparison test; Fig. 7*C*, right panel) and retreat and flight responses were induced less frequently. Retreat was observed 8% and flight 12% in the closed box. On the open platform they were observed 12% and 8%, respectively. We classified the responses mainly by the major motor responses, however, in many cases; these responses were typically followed by freezing in the case of central SC stimulation.

Movie 6. Freezing response in closed box. Pathway-nonselective stimulation (100-ms duration) of central SC induced freezing responses which last during the stimulation and for a few seconds after the stimulus offset. 10.1523/ENEURO.0271-20.2020.video.6

Thus, nonselective local stimulation of the caudo-lateral SC, representing the lower visual field, typically elicited orienting responses. Defense-like responses were evoked most frequently from local stimulation of the rostro-medial part of the SC, representing the upper visual field, however, the effects were not as context-dependent as the pathway-selective stimulation. Furthermore, freezing was the most notable feature of the nonselective stimulation of the wide area of SC.

## Discussion

### Methodological considerations

The pathway-selective activation was achieved by double vector infection in this study. Currently, pathway-selective optogenetic manipulation is achieved either by (1) transgenic lines for expression of photoactivatable opsin using a cell-type-specific promotor, or (2) injection of an opsin-expressing viral vector at the somatic location and applying stimulation to the labeled terminals, or (3) double viral vector technology: combination of a retrograde vector injection into the target area and an anterograde vector injection into the somatic location. In all these methods, additional activation of collaterals should be considered as a possible concern. In method (1), all the collaterals emerging from the target neurons would be activated. In method (2), pathway-selective activation could be achieved; however, possible antidromic activation cannot be excluded. In some cases, the possibility of antidromic activation was ruled out (Azim et al., 2014), while in other cases, the antidromic activation has been demonstrated (Saiki et al., 2018). Method (3), as in the present experiments, can be applied to any anatomically identified pathways whose cell-type-specific promotors are not identified. In this case, all collaterals of target neurons will be activated. Findings of this study were obtained by activation of the entire axonal arbors of the target neurons. In any case, it is important to know the whole axonal trajectory of the target neurons to fully understand the results of manipulation.

### Renaming of the classical crossed and uncrossed pathways

Tracing the whole axonal trajectories of both types of neurons revealed some collateral branches which were not found in the previous studies (Redgrave et al., 1987, 1988; Dean et al., 1989). Descending axons of the crossed pathway have collaterals on the ipsilateral brainstem (Fig. 3*A4*, white arrows), and those of the uncrossed pathway have collaterals on the contralateral brainstem. The possibility of diffusion of the injected vector could be excluded because projection pattern of both groups of neurons were different as shown in Figures 3, 4. Furthermore, both groups of neurons have ascending collaterals mainly on the ipsilateral side. Therefore, we decided to rename the crossed and uncrossed SC-brainstem pathway as “tectal orienting” pathway and “tectal defense” pathway, respectively.

### Primary responses to activation of the tectal orienting versus defense pathway neurons

Brief optical activation of the tectal orienting pathway neurons caused contraversive orienting-like head turn, while extended stimulation caused contraversive turn involving the whole body. In clear contrast, optical activation of the tectal defense pathway neurons elicited defense-like responses. Brief stimulation induced oblique and upward head-only turns. Extended stimulus additionally induced responses resembling retreat or flight. These patterns of defensive responses were reminiscent of those described by Redgrave and colleagues, who studied the functional status of descending projections from the SC with classical electrical and chemical stimulation techniques combined with surgical lesions (Dean et al., 1986, 1989; Sahibzada et al., 1986; Redgrave et al., 1987, 1988; King et al., 1996).

We compared the effects of activating the two pathways selectively with those obtained following nonselective optical activation of SC neurons. Stimulation of the caudo-lateral SC, in which the lower visual field is represented, evoked responses apparently similar to selective activation of the crossed pathway. Alternatively, nonselective activation of the rostro-medial SC, where the upper visual field is represented, induced responses very similar to those produced by selective activation of the tectal defense pathway. These results can be explained by considering the anatomic distribution of the cells of origin of the two descending pathways in the rodent SC, in conjunction with the ecological niche occupied by rodents (Dean et al., 1989). First, the neurons giving rise to the tectal orienting pathway are concentrated in the lateral SC (Fig. 3*B*), while those projecting to the tectal defense pathway are more prevalent medially (Fig. 4*B*). In the visual space map of the SC, the medial region corresponds to the upper visual field, while the lower visual field is represented laterally (Dräger and Hubel, 1976). For these species, their predators (birds of prey and larger mammals) most frequently approach from above. It is therefore perhaps not surprising that in these species, a specialized association between upper field visual stimuli and defensive behavior has evolved. Alternatively, most stimuli that these animals would want to locate and move toward (food and young) are typically on the ground and would appear in the lower visual field. Clearly, the evolved mechanism for this functional specialization in rodents is the differential concentration of defense-related cells in the medial upper-field portion, and cells promoting orienting behavior located laterally in lower-field regions of the SC map (Redgrave et al., 1986).

A closer look at the results of the present study, however, reveals a more nuanced picture. Functional differences were observed between the nonselective activation of SC, which most likely involved the simultaneous activation of both descending projections as well as activation of the superficial layers (SCs). When the central SC was activated with an optic fiber with large diameter, freezing responses were observed more frequently than the localized stimulations. Optical stimulation with a large fiber could conceivably correspond to the sudden appearance of a large object covering much of the animal’s visual field, similar to the looming stimulus. Such a stimulus event would be unusual in nature, and it would be likely that both orienting-approach and defense-avoidance systems in the SC would have been activated simultaneously. If so, unresolved competition between them could mean that freezing would be the most adaptive response. This hypothesis could be tested by systematically changing the diameter of photostimulation, which itself is technically challenging but could be tried in future. An alternative possibility for the prevalence of freezing by central SC stimulation could be that the stimulation may also have activated other SC outputs. Recent studies by Wei et al. (2015), and Shang et al. (2015), reported freezing following selective activation of cell groups in the SCs. This response was ascribed to indirect activation of amygdala by visual inputs routed through the SC.

### Shortest pathways for the motor outputs

Here, we were able to show that activation of each descending pathway evoked EMG responses in the dorsal neck muscles ∼3–5 ms after optically induced spiking in the SCd (Fig. 5). The descending routes of communication were confirmed when critical relays in the brainstem reticular formation were inactivated, optically elicited EMG responses abolished. This result is consistent with previous studies in cats that showed the shortest pathway from the SC to neck motoneurons was mediated by reticulospinal neurons in the PMRF projecting bilaterally to neck motoneurons (Anderson et al., 1971; Iwamoto et al., 1990; Isa and Sasaki, 2002). In case of the defense pathway, the short latency EMG responses could be explained by a tri-synaptic linkage from the SCd to neck motoneurons presumably mediated by neurons in the MRF and reticulospinal neurons in the PMRF as suggested previously in the cat by Alstermark et al. (1992). It might be surprising that inactivation of the brainstem reticular formation totally eliminated the neck muscle responses on both sides. This would be primarily because the major portion of the SC effects on the neck muscle activity is mediated by the uncrossed and crossed pontine and medullary reticulospinal neurons, not by the direct tectospinal tract. This speculation would be supported by observation in Figure 3*A7*, showing the presence of relatively few tectospinal axons compared with the large number of PDB axons visible in the brainstem (Fig. 3*A5*).

### Long-latency, context-dependent responses to the tectal defense pathway activation and their possible anatomic basis

In addition to the robust short latency responses induced via oligosynaptic pathway to motoneurons (see above in Discussion section), we also observed longer latency defense-like responses which appeared to be context-dependent. It is likely that context-dependent effects of defense-like responses originated from the forebrain regions which may be supplied with information carried by ascending collaterals from the tectal defense pathway neurons. An important advantage of the current pathway-selective activation procedures was that the entire network of axonal projections, including collaterals, of activated neurons could be traced. The tectal defense pathway neurons project ascending branches widely to midbrain and thalamic nuclei and descending branches toward a variety of targets in the pons and medulla, some of which are different from targets of the orienting pathway (Figs. 3*C*, 4*C*). It is likely that some of the target regions of defense pathway neurons may be associated with different components of the elicited responses. For example, the SC-PPN/CnF projections might be involved in the locomotor aspects of defense-like behaviors, because PPN and CnF have been considered as the mesencephalic locomotor region (Shik et al., 1966). Dense projection to PoT (posterior thalamic nucleus, triangular) might be related to aversive itch or pain perception (Lipshetz et al., 2018). Other target regions of the thalamus have connections to the cerebral cortex, limbic system (Doron and Ledoux, 2000), and basal ganglia (Beckstead, 1984). Return connections from these forebrain regions to the SC and other brainstem targets could be the means whereby affective and cognitive influences might modify the motor component of defensive responses to visual threat. Therefore, it is particularly interesting that the ascending collaterals from the SCd may reach the amygdala, because if such efference copy signal of the innate defense response activates the amygdala, it would indicate the existence of retrospective activation system of fearful emotion succeeding the motor responses.

The trajectories of tectal orienting pathway neurons partly correspond to those of presaccadic bursting neurons found in cats (Grantyn and Grantyn, 1982) and squirrel monkeys (Moschovakis et al., 1988a,b). In addition to projection to the contralateral PMRF, they also possessed ipsilateral projections to SNc, mRt, PPN, and a part of the PRMF, in common with the tectal defense pathway, which was missed in the previous study (Redgrave et al., 1987, 1988; Dean et al., 1989).

It is now clear that there are multiple output channels from the SC, both SCs and SCd, which mediate approach orienting and defense-related signals. The precise nature of these responses can be modified by contextual cues. The role played by each channel in different aspects of the adaptive responses to appetitive and threatening visual stimuli, and how they can be modified by top-down influences, represent important goals for future studies.

## Synthesis

Reviewing Editor: Bradley Postle, University of Wisconsin Decisions are customarily a result of the Reviewing Editor and the peer reviewers coming together and discussing their recommendations until a consensus is reached. When revisions are invited, a fact-based synthesis statement explaining their decision and outlining what is needed to prepare a revision will be listed below. The following reviewer(s) agreed to reveal their identity: Tiago Branco.

Here is the full text of R2’s review. Please note that there not expectation that you would carry out the proposed additional experiment involving ChR2 expression.

This manuscript uses viral tracing and optogenetics to investigate the anatomy and behavioural role of crossed and uncrossed projections of the Superior Colliculus (SC). The main advances of this work are 1) refining our knowledge of the anatomy of these two projections, including the description of new collaterals and the observation that both pathways include crossed and uncrossed axons; 2) observing orienting and defensive responses that scale with the intensity of optogenetic stimulation; 3) identifying behavioural responses elicited by stimulation of the uncrossed pathway as depending of the spatial properties of the arena - flight when the arena is small and has wall, retreat in a larger arena with no walls.

I think that experiments are performed to a good standard and well designed. The use of the viral strategy for targeting the two pathways is a welcome refinement that generated anatomical information useful for the field. The most interesting advance of the behavioural experiments is the context-dependency of the stimulation of the uncrossed pathway. While it is unclear why this happens, it does provide hypotheses that can be addressed in future studies. I have a couple of questions and comments:

1) The non-specific activation of SC is useful for linking the two pathways to the visual field, even if the results are an minor extension of the previous literature. The interesting result here is that stimulation of the rostro-medial SC can elicit freezing, which was not observed with activation of the uncrossed pathway. As the authors point out, it is hard to interpret why freezing is observed in this condition and there are several possible explanations. One hypothesis is that freezing arises from activation of a large area of the visual field through the superficial SC - this is very interesting and could be tested directly by expressing ChR2 in the rostro-medial superficial SC, and then gradually increasing the diameter of the stimulation to test if freezing is induced and whether there is a diameter threshold for this.

2) I might have missed it, but I didn’t see statistical tests for the change in behaviour proportions for different stimulation parameters. Also, there are no statistical test for an effect of stimulus. This is particularly important for head and body turns, as these are behaviours that mice do spontaneously at a fairly high frequency - e.g.: what is the probability that a movement classified as head or body turn is observed without stimulation, ideally matched to the conditions where a stimulus would have been delivered.

3) Does the direction of flight with optogenetic stimulation of the uncrossed pathway depend on the distance or orientation to the wall in the small environment?

4) Please show an example video for a freezing response
